# Developing a health and human rights training program for french speaking Africa: lessons learned, from needs assessment to a pilot program

**DOI:** 10.1186/1472-698X-9-19

**Published:** 2009-08-24

**Authors:** Philippe Chastonay, Axel Max Klohn, Véronique Zesiger, Franziska Freigburghaus, Emmanuel Kabengele Mpinga

**Affiliations:** 1 Institute of Social and Preventive Medicine, Department of Community Health and Medicine, Faculty of Medicine, University of Geneva, Geneva, Switzerland; 2 Swiss Agency for Development and Cooperation, Bern, Switzerland; 3 Swiss School of Public Health plus, Zurich, Switzerland

## Abstract

**Background:**

The importance of human rights education has widely been recognized as one of the strategies for their protection and promotion of health. Yet training programs have not always taken into account neither local needs, nor public health relevance, nor pedagogical efficacy.

The objectives of our study were to assess, in a participative way, educational needs in the field of health and human rights among potential trainees in six French-speaking African countries and to test the feasibility of a training program through a pilot test. Ultimately the project aims to implement *a health and human rights training program most appropriate to the African context*.

**Methods:**

*Needs assessment *was done according to four approaches: Revue of available data on health and human rights in the targeted countries; Country visits by one of the authors meeting key institutions; Focus group discussions with key-informants in each country; A questionnaire-based study targeting health professionals and human rights activists.

*Pilot training program*: an interactive e-learning pilot program was developed integrating training needs expressed by partner institutions and potential trainees.

**Results:**

Needs assessment showed high public health and human rights challenges that the target countries have to face. It also showed precise demands of partner institutions in regard to a health and human rights training program. It further allowed defining training objectives and core competencies useful to potential employers and future students as well as specific training contents.

A pilot program allowed testing the motivation of students, the feasibility of an interactive educational approach and identifying potential difficulties.

**Conclusion:**

In combining various approaches our study was able to show that training needs concentrate around tools allowing the identification of basic human rights violations in the health system, the analysis of their causes and coordinated responses through specific intervention projects.

## Background

The importance of human rights education for its intrinsic value has been high on the UN agenda for years, culminating in a resolution proclaiming a decade of human rights education and proposing an action plan [1,2]. The importance of human rights education has also widely been recognized as one of the strategies for their protection and promotion of health [3,4]. Its importance has further been stressed, be it in the field of the health of the population or in the particular context of health care, where many basic human rights violations are still prevalent [5,6].

Indeed human rights education programs targeting health professionals have been implemented in many places around the world over the past decade [5,7-9]. Yet too often such programs have ignored educational recommendations stressing the need of public health relevance and pedagogic efficacy of the programs [10,11]. Taking into account those two dimensions, i.e. public health relevance and pedagogic efficacy, implies prior needs assessment among target communities and actors [12,13].

The present paper presents, in the context of a project focusing on human rights education in the health sector in French speaking African countries supported by the Swiss Agency for Development and Cooperation [14], the process of developing and implementing a training program on health and human rights, starting from the local needs assessment to the evaluation of a curriculum implemented as a pilot program.

## Methods

The project was accepted by the Institutional Review Boards of the Institute of Social and Preventive Medicine of the University of Geneva and of the Swiss Agency for Development and Cooperation.

### A. Needs assessment was done through 4 different approaches

#### 1. A review of available data on Health and Human Rights in the targeted countries

UN agency based reports and NGO's reports [15-17] were checked on health indicators and human rights situations. Major issues were brought together in a synthetic, yet not exhaustive way, in order to establish a global framework of possible learning objectives for a Health and Human Rights course.

#### 2. Country visits by one of the authors (EKM)

Country visits were done through two field missions 6 months apart in 2007. Visited countries included Chad, Burkina Faso, Mali, Ivory Coast and Congo Brazzaville. Out of security reasons a delegation from the DR of Congo was met at Brazzaville (sporadic fighting among rival political factions in Kinshasa in November 2007). Professional associations (public health and human rights associations) were visited and semi-directed interviews were made with board members [18]. Representatives of the Ministries of Health or Education were also met in order to facilitate the implementation of the considered pilot program.

#### 3. Focus group discussions with key informants in each country

Focus group discussions were held with key-informants (discussions held with 73 key-informants, organized as multiple small-group sessions) selected by the professional associations in each country. A total of 9 focus groups were organized (2-hour session each). Each group's work was organized according to standard procedures [19,20]: special attention was given to the constitution of the group (a mixture of health professionals and human rights activists); a series of open-ended questions addressing possible educational objectives in the field of health and human rights were presented to each group; key issues raised in a first round of discussion were reported on a flip chart and further discussed in a second round with the aim of setting priorities.

Discussed topics included core human rights competencies in the field of public health, public health competencies of use to promote human rights, public health issues that might benefit from a human rights approach, possible course contents. Discussions were not taped, but one of the researchers (EKM) took extensive notes. Analysis was done according to standard focus group content analysis procedures [21].

#### 4. A questionnaire-based study

The questionnaire [additional file 1] was developed in a similar way as the list of competencies developed for ethical public health practice [22]: the core competencies of public health, as established by the Council on Linkages between Academia and Public Health Practice served as a starting point [23]; the Public Health Code of Ethics was also consulted [24]; in the process, competencies, as suggested in UN human rights documents, were also integrated [25], as well as tasks commonly developed in the field [26]. The lists were discussed within the research team and submitted for validation to the visited country professional associations as well as to representatives of international agencies. The questionnaire included items (on a Likert scale) on knowledge, attitudes and tasks in the field of health and human rights. The final questionnaire was tested on a set of health professionals taking a community health course as well as a group of professionals taking a course on discriminations, health and human rights.

The questionnaire was addressed from spring to autumn 2007 to health professionals and human rights activists, who had been identified by local professional associations on a random basis in each target country.

The questionnaire also included several items on most appropriate educational approaches (from frontal lectures to student-centered community projects).

Data analysis of the questionnaire study was done with EpiInfo/EpiData [27] and the R Open Source software [28].

### B. The educational approaches tested in the pilot program

Once data from the needs assessment had been analyzed, an Internet pilot program was developed based on the collected data from the focus groups and the questionnaire study. The content was first conceived by the authors and than adapted after the input of key-informants of partner institutions. The program was tested during spring 2008 among 20 professionals from the participating countries (registration on a voluntary basis once selected by the local association).

The program included specific health and human rights Internet interactive seminars given by public health and human rights experts over the KM4PH network of the World Health Organization [29], case-studies to be analyzed and commented as well as reports to be written by the students; furthermore it included a thesis based on a community project.

Topics treated included identified health and human rights issues such as child labor, discrimination and violence against women, discrimination and violence against persons with mental health problems, torture (see bellow).

Evaluation focused on student participation, student satisfaction, student achievement as perceived by tutors and implemented (planned to be implemented) community research/action projects.

The project was supported by the Swiss Agency for Development and Cooperation, which facilitated local contacts and made local logistic support readily available.

## Results

### Needs assessment regarding a course on health and human rights

The data on Health and Human Rights in the *targeted countries *was presented in a synthetic way in the Vision of Humanity Report [17]. It is summed up in Table 1. It appears that the targeted countries all have difficult records on human rights and public health development as per the Global Peace Index.

**Table 1:** Human rights an health indicators of target countries according Vision of Humanity Global Peace Index 2008

Country	IMR	LEB	GDP/hab	PIN	SOCL	ROHR	Global Peace Index Ranking among 140 countries
Chad	123.9	44	619	3.8	3.2	4	135

DR Congo	129	44	141	4	2.4	5	128

Ivory Coast	118	46.2	928	4.25	3.8	4	122

Congo	81	52.8	2.250	3.5	3.8	2	117

Mali	120	48.6	490	4	6.5	2	99

Burkina Faso	96	48.5	421	2.5	4.4	5	81

*In comparison e.g. Denmark*	4	77.8	50.859	1	9.7	1	2

IMR: infant mortality rate (first year mortality per 1000 living birth)

LEB: life expectancy at birth

GDP: gross domestic product per inhabitant in $ US

PIN: political instability (from 1: low; to 5: high)

SOCL: state of civil liberties (from 10: good; to 1: bad)

ROHR: respect of human rights (from 1: good; to 5: bad)

The country visits allowed in-depth conversations with the heads of the concerned *professional associations *(health/human rights). The list of visited associations appears in Table 2. The main expressed concern was a true partnership in planning, implementing and evaluating the announced training program. Furthermore the need for developing a health and human rights training program of strong public health relevance and high educational efficacy were stressed by the various consulted associations.

**Table 2:** Institution representatives met during the training needs' assessment field-missions

	Burkina Faso	Chad	Congo	DR Congo	Ivory Coast	Mali
National Committee/Association/Observatory of Human Rights	+	+	+		+	+

Medical Association		+	+	(+)	+	+

Ministry of Health and/or Human Rights/and/or Education	+	+	+		+	+

University and/or Faculty of medicine	+	+	+		+	+

National Institute of Public Health		+			+	

Civil Society Movement and/or Counsel	+	+	+	+	+	+

Lawyers'/Judges' Associations		+	+	+	+	

Women's Associations	+	+	+		+	+

Press Association	+	+	+		+	+

Associations against torture	+		+		+	+

Counsel of nurses	+		+	(+)	+	

Patients' Associations		+	+		+	

Amnesty International	+				+	

The *focus group *discussions yielded specific training demands summarized in Table 3. It appears that basic public health competencies for health professionals and human rights activists, such as needs assessment tools, project management methodology, project impact assessment methods, health and human rights lobbying strategies, are needed.

**Table 3:** Consensus around priority educational objectives of a health and human rights training program: Focus group discussions with key informants (n: 73)

Ranking	*Priority educational objectives for graduates of the program: participants should be able to*
Objective 1	*lobby in favor of the respect of human rights of individuals and groups of population*

Objective 2	*master negotiating skills in order to interact with State institutions and agencies*

Objective 3	monitor the implementation of human rights strategies in the health sector*

Objective 4	*collect and analyze public health data in order to prevent and promote human rights in the community*

*Objective 5*	*plan, implement and evaluate public health and human rights projects*

*as defined in Human Rights, Health and Poverty reduction strategies WHO/ETH/HDP05, 2005

The results from the *questionnaire-based study *targeting health professionals and human rights activists who had been identified by local professional associations are presented in Table 4. The participation rate was 66% (238 respondents/360 selected participants).

**Table 4:** Expected competencies to be acquired through a health and human rights training program in a questionnaire-study among health professionals and human rights activists (n: 238)

Core competencies	*% mentioning the competency as very important or extremely important (n: 238)*
By the end of the program participants should be able to:	
*• Establish the diagnosis of a health and human rights critical situation*	
- Determine the health needs and priorities of the communities	87.8
- Determine the needs in protecting basic human rights related to health	87.8
- Identify the violations of basic human rights in the health system	84.5
- Identify the risk factors of major public health problems	81.5
*• Use appropriate methods and show appropriate attitudes*	
- Work in the spirit of justice and equity	82.2
- Work with mechanisms of human rights protection	79.8
- Use public health data collection and analysis methods	75.6
*• Act to promote and protect human rights*	
- Prevent the violations of basic human rights in the health sector	79.4
- Use human rights tools in health promotion programs	73.9
- Rehabilitate victims of human rights violations	70.2
- Plan and evaluate public health programs integrating human rights	70.2

The respondents to the questionnaire put strong emphasis on core public health competencies such as health needs assessment tools for public health challenges and human rights violations (>85%), but also on insight understanding (better knowledge) of risk factors of basic human rights abuse in the health system and of major public health problems (>80%) as well as on appropriate attitudes to develop, i.e. justice and equity (>80%). Statistical analysis showed no significant differences when responses were analyzed according to age, sex, religion, marital status or professional experience.

### The educational approaches tested in the pilot training program

The pilot training program focused on a distance-learning, yet interactive, approach.

It consisted in:

- interactive e-teaching seminars with *content *focusing on specific health and human rights topics that had been identified as relevant in an African context (child labor, discrimination and violence against women, discrimination and violence against persons with mental health problems, torture) as well as more methodological seminars (human rights legislation and international mechanisms of health protection); done over the KM4PH network of WHO [29] with ASP hosted tele-collaboration software, it allowed interactive sessions between participants and public health and human rights experts (conferences, written on line questions, oral questions);

- case-studies that students had to work and report on related to common health and human rights challenges in many African countries (mother and child health, genital mutilation, non registration of birth and health and human rights consequences on the long run, access to food, water and sanitation, etc. were done as assignment with a deadline to respect; individual feedback was given by teaching staff checking for plagiarism (several cases), relevance of bibliography (observed difficulties of coherent bibliographic research with adequate key notes) and intrinsic coherence;

- a community based health and human rights project, to be identified, planned and implemented by each student, that addresses a relevant health and human rights problem of the community, such as:

◦ Prevention of violence against women in the province of North-Kivu of the DR Congo,

◦ Violence against children: a public health problem in Ivory Coast,

◦ Child labor: the situation in Abidjan, Ivory Coast,

◦ Health in detention in N'djamena, Chad,

◦ Critical analysis of the legal and political framework of adolescent reproductive health in Burkina Faso.

The evaluation procedures of the pilot training program showed a high level of student satisfaction (>80%): the program met expectations and training needs, work with the case studies promoted acquisition of specific public health and human rights tools, the lectures clarified concepts related to discrimination, health and human rights and gave inside information on specific health and human rights topics, the interactive approach as well as the personal work were a source of motivation.

The evaluation also showed a high level of motivation of most participants (on-line attendance despite difficulties with Internet connections, questions raised during interactive lectures, meeting deadlines for assignments, quality of the analysis of case studies). Yet the evaluation showed difficulties and points to be improved, such as constraints in meeting deadlines, restricted Internet connectivity, time consuming individual assessment and feed-back, meeting students expectations, risk of plagiarism in assignments, heterogeneity of student body, choosing topics to be discussed related to local issues, request of residential sessions, demand for scholarships, expectation of accessible databases on health and human rights, etc.

## Discussion

Identified training needs by partner institutions and professionals were consistently around core competencies in public health and basic knowledge and know-how in the field of human rights. A high homogeneity in responses was observed as well among focus group participants as the questionnaire-study respondents of the questionnaire study. Indeed, there appeared a strong demand for tools allowing: (a) the identification of basic human rights violations in the health system; (b) the analysis of their causes; (c) coordinated responses through specific intervention projects.

This strong demand for basic public health planning and management tools might be related to the well recognized importance of project management as well in public health as in the field of human rights, as illustrated by the core competencies in health and human rights of nursing training programs in South Africa [11] or in emergency and natural disaster training programs [30].

Other competencies, such as competencies in social mobilization or specific research tools, have not been identified as priority training objectives, neither in the focus groups, nor by the questionnaire-study respondents. Regarding the social mobilization it might well reflect the feeling of the professionals that "they have what it takes" to perform well. Regarding research, it still is a neglected field in the domain of health and human rights [31] and few institutions are active in this area in Africa [32]. Of course training needs in various domains vary depending on the target populations, yet the opinion of these target populations is important in defining training objectives and contents [33,34].

Developing the pilot training program appeared as a complex, yet stimulating process. It requested close collaboration with the partner institutions: this was a permanent networking challenge; all the more the program was supported by a WHO run network, the Knowledge Management for Public Health network [35].

So what are the lessons learned?

First, needs assessment gives valuable information on the local situation and on the expectations of partner institutions: indeed the Global Peace Index for targeted countries does show that public health and human rights challenges do exist in these countries. Though it takes some effort to achieve, it allows fixing the program into a local context, thus increasing the programs public health relevance [36].

Second, among potential partners, members of visited human rights and public health institutions stressed the need for a true partnership, i.e. taking into account, when developing a training program, their expectations, priorities, limits and realities. It appears that the approach facilitates partnership and insures support to the training program on a long-term basis [37].

Third, consensus can be obtained when discussing with potential employers of future trainees on what competencies such a program could/should focus on and what levels of intervention such a program could/should address [38]. Obtaining adhesion of employers to a training program has been shown as key to high impact of the program as measured by community outcomes [39].

Fourth, a list of specific educational objectives, through questioning potential trainees, can be obtained, which might reflect grass-root needs, thus ultimately insuring high public health relevance to the program [40].

Fifth, the pilot program, based on the needs assessment allowed to experiment various educational approaches, such as interactive lectures, question-answer debates, case-studies, e-learning, community project planning, educational approaches most participants had not been familiar with, being more used to formal lectures, yet approaches that have been reported as highly effective in terms of reaching specific educational objectives and setting the stage for life-long learning [41]. The pilot project showed that these approaches, though difficult at times due to logistic problems (access to Internet, computer configuration issues), to deadlines imposed by training staff, to self-discipline of students, were appreciated by students and even seemed to act as a motivating factor.

Last, the pilot project allowed experimenting the potential development of community projects by the student, with specific community outcomes to be assessed in the future, thus strengthening the student-centered approach [42].

But difficulties appeared as quite real, some being unexpected such as plagiarism, putting the teaching-team under pressure and showing some limits of the case-study approach. Indeed plagiarism appears as a hot issue in education worldwide [43] and handling it properly is not an easy task [44]. However, the observed plagiarism rates were in line with generally reported values, and along the course the students showed notable improvements in their writing and citation practices. Another difficulty was related to the heterogeneity of the student body, which is a well-known challenge in continuous adult education [45]: it can partly be resolved through personal assignments of different difficulties according to the students' level, thus designing a more student-centered program [41]. And then there were logistic problems such as access to Internet, which brought us to negotiate an access for the students of our "future" health and human rights program to the Campus Virtuel of the Francophonie [46].

## Conclusion

The study aimed to assess in a participative way educational needs in the field of health and human rights among potential trainees in six French-speaking African countries and to test the feasibility of a training program through a pilot program. Ultimately the project aims to implement a health and human rights training program most appropriate to the African context.

In combining various approaches our study was able to show that training needs concentrate around tools allowing the identification of basic human rights violations in the health system, the analysis of their causes and coordinated responses through specific intervention projects.

Furthermore the pilot training program allowed identifying potential technical problems, cultural and pedagogical hurdles, as well as testing educational approaches and course contents.

Going trough a needs assessment procedure and a pilot program when planning a new curriculum in specific, and perhaps unfamiliar, settings might well keep the designers and leaders of a program from straightforward failure: indeed the approach fosters public health relevance and educational effectiveness. Thus this approach might well be of great value on the long run.

## Competing interests

The authors declare that they have no competing interests.

## Authors' contributions

EKM and PC conceived the project and obtained the support of the Swiss Agency for Cooperation and Development; they were in charge of interpreting the collected data and writing the first draft of the manuscript. AMK managed and analyzed the data and revised thoroughly the manuscript. VZ and FF revised the project and the manuscript. EKM developed the network and collected the data. All authors approved the final manuscript.

## Pre-publication history

The pre-publication history for this paper can be accessed here:

http://www.biomedcentral.com/1472-698X/9/19/prepub

## Supplementary Material

Additional file 1 **Afro Study**. The file contains the questionnaire addressed to health professionals and human rights activists. Click here for file
