# Supplementary material for: Developing a health and human rights training program for french speaking Africa: lessons learned, from needs assessment to a pilot program
Source: BMC Int Health Hum Rights. 2009 Aug 24;9:19. doi: 10.1186/1472-698X-9-19 (PMC2745355; doi:10.1186/1472-698X-9-19)
Supplement: Additional file 1 — Afro Study. The file contains the questionnaire addressed to health professionals and human rights activists. [file 1472-698X-9-19-S1.doc]

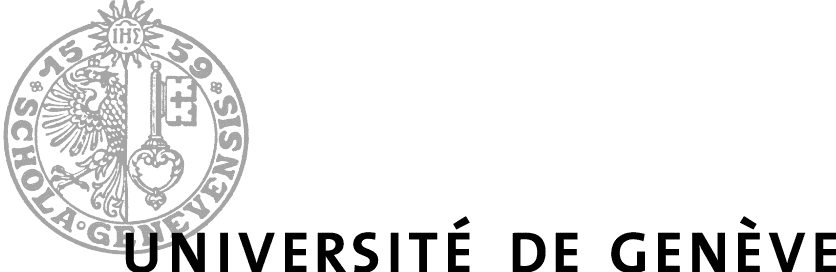


FACULTE DE MEDECINE

**ETUDE DE BESOINS EN FORMATION DES FORMATEURS SANTE DROITS HUMAINS - 2007**

Dans le cadre du Projet de Formation des Formateurs en Santé et Droits de l’Homme de l’Université de Genève et de la Direction Suisse d’Aide à la coopération, le présent questionnaire vise à identifier les besoins en formation tels que ressentis par les défenseurs des droits humains et les professionnels de santé. Les résultats de ce questionnaire permettront de concevoir les modules de cette formation, de choisir des méthodes adaptées, de définir les modalités pédagogiques et l’organisation matérielle d’un tel programme.

L’exploitation des données de ce questionnaire se fera de manière anonyme, l’Université de Genève s’engage à respecter la confidentialité et veillera à l’usage non abusif de ces données.

**CONSIGNES**

1. Lisez attentivement l’ensemble de questions
2. Répondez à la série de questions de manière progressive
3. Pour les questions II à VI, merci de donner votre opinion à chaque proposition de réponse.

Merci.

1. **VOS CARACTERISTIQUES**
2. Pays ……………………………………………………………………………………………………………………………..
3. Ville………………………………………………………………………………………………………………………………
4. Province / District……………………………………………………………………………………………………………….
5. Sexe Masculin ⁪ Féminin ⁪
6. Age en années révolues………………………………………………………………………………………………………….
7. Profession

Professionnel de santé ⁪ Médecin ⁪ Infirmier (e) ⁪ Pharmacien(ne) ⁪ Dentiste ⁪ Vétérinaire ⁪ Autres

Défenseurs de droits humains ⁪ Magistrat ⁪ Avocat ⁪ Journaliste ⁪ Sciences humaines-sociales ⁪ Autres

1. Expérience professionnelle en années après obtention du diplôme de base …………………………………………………..ans
2. Confessions religieuses / spiritualités

⁪ Chrétienne ⁪ Musulmane ⁪ Juive ⁪ Animiste ⁪ Athée ⁪ Sans ⁪ Autres (préciser)………………………

1. Etat Civil

⁪ Célibataire ⁪ Marié(e) ⁪ Veuf (ve) ⁪ Divorcée

1. **Fonctions Importantes dans les activités de la Santé Publique et des Droits de l’homme (ddh)**

Quelles sont, selon vous, les **fonctions et tâches**  jugées importantes dans les activités de défense et promotion de droits de l’homme d’une part et celles de santé publique d’autre part. **Pour chaque proposition, merci d’indiquer cette importance en choisissant la valeur correspondante (1 : sans importance à 4 extrêmement important ; cocher le 9 si vous ne savez pas).**

| Fonctions et activités | 1. Sans Importance | 2. Important | 3. Très important | 4. Extrêmement  Important | 9. Ne sait |
| --- | --- | --- | --- | --- | --- |
| 1. Détermination des besoins de protection des ddh |  |  |  |  |  |
| 2. Détermination des besoins de santé publique |  |  |  |  |  |
| 3. Identification des priorités de protection/ santé |  |  |  |  |  |
| 4. Planification des activités |  |  |  |  |  |
| 5. Communication interne / externe |  |  |  |  |  |
| 6. Négociation avec les acteurs concernés |  |  |  |  |  |
| 7. Gestion des structures et organisations |  |  |  |  |  |
| 8. Utilisation des mécanismes de protection |  |  |  |  |  |
| 9. Evaluation des programmes et activités |  |  |  |  |  |
| 10. Intervention auprès des autorités |  |  |  |  |  |
| 11. Réhabilitation des victimes |  |  |  |  |  |
| 12. Mobilisation sociale santé –droits de l’homme |  |  |  |  |  |
| 13. Constitution et utilisation des réseaux de soutien |  |  |  |  |  |
| 14. Coordination des activités |  |  |  |  |  |
| 15. Formation aux stratégies et techniques |  |  |  |  |  |
| 16. Recherche en Santé /droits de l’homme |  |  |  |  |  |
| 17. Collaboration avec les structures nationales/ Intern. |  |  |  |  |  |
| 18. Participation aux activités nationales/ internationales |  |  |  |  |  |

1. **CONNAISSANCES NECESSAIRES DANS LE DOMAINE DE LA SANTE ET DES DROITS DE L’HOMME ( ddh)**

Quelles sont **les connaissances** nécessaires à un meilleur accomplissement de ces tâches et fonctions. **Pour chaque proposition, merci d’indiquer cette nécessité en choisissant la valeur correspondante (1 : Pas nécessaire, à 4 : Extrêmemen**t **nécessaire ; cocher le 9 si vous ne savez pas**).

| Connaissances | 1. Pas Nécessaires | 2. Nécessaires | 3. Très nécessaires | 4. Extrêmement  nécessaires | 9. Ne sait |
| --- | --- | --- | --- | --- | --- |
| 1. La Théorie générale sur les droits de l’homme |  |  |  |  |  |
| 2. La Théorie générale sur la santé publique |  |  |  |  |  |
| 3. Les Systèmes et mécanismes de protection des ddh |  |  |  |  |  |
| 4. Les Méthodes de collecte et analyse des données |  |  |  |  |  |
| 5. Les Déterminants et facteurs de risque en droits de l’homme |  |  |  |  |  |
| 6. Les Déterminants et facteurs de risque en santé publique |  |  |  |  |  |
| 7. Les Principaux problèmes de santé Internationale |  |  |  |  |  |
| 8. La Pédagogie pour adultes |  |  |  |  |  |
| 9. Les Problèmes Internationaux des droits de l’homme |  |  |  |  |  |
| 10. Les Violations des droits de l’homme dans les systèmes de santé |  |  |  |  |  |
| 11. Les Stratégies de communication sociale pour les ddh |  |  |  |  |  |
| 12. Le Monitoring de l’état de santé et des ddh |  |  |  |  |  |
| 13. Les Systèmes judiciaires nationaux et la santé publique |  |  |  |  |  |
| 14. Les Institutions Nationales de protection des ddh |  |  |  |  |  |
| 15. La Planification des projets de santé et des ddh |  |  |  |  |  |
| 16. Les Dimensions culturelles de la santé et des ddh |  |  |  |  |  |
| 17. Les Techniques de négociation pour les ddh |  |  |  |  |  |

1. **Aptitudes à développer pour un meilleur exercice des fonctions et activités de protection et promotion de la santé et de droits de**

**l’homme.**

Selon vous, quelles sont les aptitudes importantes à un meilleur accomplissement des tâches et fonctions de protection et promotion de

la santé et des droits de l’homme. **Pour chaque proposition, indiquer l’importance de chaque aptitude en choisissant sa case**

**correspondante (1 : sans importance à 4 Extrêmement important ; cocher le 9 si vous ne savez pas).**

| Aptitudes à | 1. Sans Importance | 2. Important | 3. Très important | 4. Extrêmement  Important | 9. Ne sait |
| --- | --- | --- | --- | --- | --- |
| 1. Prévenir les risques des atteintes aux ddh en santé publ. |  |  |  |  |  |
| 2. Utiliser les ddh comme outil de promotion santé |  |  |  |  |  |
| 3. Utiliser les données sanitaires promotion ddh |  |  |  |  |  |
| 4. Elaborer, mettre en œuvre et évaluer des projets |  |  |  |  |  |
| 5. Rechercher, critiquer, analyser et publier les informat. |  |  |  |  |  |
| 6. Vulgariser les droits de l’homme |  |  |  |  |  |
| 7. Participer activement aux activités des réseaux |  |  |  |  |  |
| 8. Evaluer les pair(e)s et s’auto-évaluer |  |  |  |  |  |
| 9. Diagnostiquer l’état des ddh/ situation de santé |  |  |  |  |  |
| 10. Synthétiser les informations complexes |  |  |  |  |  |
| 11. Communiquer oralement et argumenter ses avis |  |  |  |  |  |
| 12. Utiliser les ressources locales et internationales |  |  |  |  |  |
| 13. Identifier et Utiliser les sources de financement |  |  |  |  |  |
| 14. Autres (préciser)  ………………………………………………………..  …………………………………………………………  ………………………………………………………… |  |  |  |  |  |

1. **Attitudes à développer dans la protection et la promotion des droits de l’homme et de la santé**

Quelles seraient, selon vous, les attitudes indispensables à acquérir ou à développer à la suite d’une formation sur la santé et les droits de l’homme.

**Pour chaque proposition, merci d’indiquer l’importance que vous accordez à chaque attitude en choisissant entre**

**1. pas indispensable à 4. extrêmement indispensable, cocher le 9 si vous ne savez pas**

| Attitudes | 1. Pas Indispensable | 2. Indispensable | 3. Très Indispensable | 4. Extrêmement Indispensable | 9. Ne sait |
| --- | --- | --- | --- | --- | --- |
| 1. L’humilité |  |  |  |  |  |
| 2. L’esprit de Justice |  |  |  |  |  |
| 3. La droiture |  |  |  |  |  |
| 4. L’équité |  |  |  |  |  |
| 5. La responsabilité |  |  |  |  |  |
| 6. L’esprit de sacrifice |  |  |  |  |  |
| 7. La recherche de la reconnaissance |  |  |  |  |  |
| 8. La compassion |  |  |  |  |  |
| 9. Le sens d’organisation |  |  |  |  |  |
| 10. L’abnégation |  |  |  |  |  |
| 11. La tolérance |  |  |  |  |  |
| 12. Le dialogue |  |  |  |  |  |
| 13. La persuasion |  |  |  |  |  |
| 14. L’amour de la vérité |  |  |  |  |  |
| 15. Le don de soi |  |  |  |  |  |
| 16. Autres (à préciser)  ………………………………………………………  ………………………………………………………. |  |  |  |  |  |

1. **Publics Cibles**

Selon vous, quels seraient les personnes, institutions ou structures concernés par la formation à la santé et aux droits de l’homme.

**Pour chaque proposition, merci d’indiquer cette nécessité en choisissant entre (1 : pas concernés à 4 : extrêmement concernés).**

| Publics Cibles | 1. Pas concernés | 2. concernés | 3. Très concernés | 4. Extrêmement  concernés | 9. Ne sait |
| --- | --- | --- | --- | --- | --- |
| 1. Les professionnels de santé |  |  |  |  |  |
| 2. Les défenseurs des droits de l’homme |  |  |  |  |  |
| 3. Les autorités judiciaires, administratives et politiques |  |  |  |  |  |
| 4. Les parlementaires |  |  |  |  |  |
| 5. Les associations des patients |  |  |  |  |  |
| 6. Les groupes de la société civile |  |  |  |  |  |
| 7. Les autorités confessionnelles / religieuses |  |  |  |  |  |
| 8. Les Assureurs |  |  |  |  |  |
| 9. Les formateurs en sciences de la santé et action sociale |  |  |  |  |  |
| 10. Les Agences d’aide à la coopération et au dévelop. |  |  |  |  |  |
| 11. Les Ongs humanitaires |  |  |  |  |  |
| 12. Les Guérisseurs, marabouts et autorités traditionnelles |  |  |  |  |  |
| 13. Les chercheurs en sciences sociales et santé |  |  |  |  |  |
| 14. Les journalistes et hommes des médias |  |  |  |  |  |
| 15. Autres (à préciser)  ………………………………………………………  ……………………………………………………….  ………………………………………………………..  …………………………………………………………. |  |  |  |  |  |

1. **MODALITES PEDAGOGIQUES**

**7.1. Méthodes de travail**

Parmi les méthodes de travail suivantes, choisissez celle que vous souhaiteriez dans le cadre d’un programme de formation des formateurs

en santé et droits humains. *(une seule réponse)*

1. ⁪ Les exposés magistraux 2. ⁪ L’étude des cas 3. ⁪ L’Apprentissage par résolution des problèmes 4. ⁪ Les séminaires ateliers

5. ⁪ Une combinaison de ces différentes méthodes 6. ⁪ Autres (à préciser)…………………………………………………

7. ⁪ Sans opinion

- 1. **Lieux et modalités d’organisation de la formation**

Pour être davantage bénéfique aux participants, une telle formation devrait être organisée (*plusieurs réponses possibles*) :

1. ⁪ localement 2. ⁪ à l’étranger en particulier auprès des institutions internationales des ddh et de la santé

3. ⁪ localement et à l’étranger de manière alternée 4. ⁪ uniquement par échanges à l’aide de nouvelles technologies de l’information

5. ⁪ sans opinion

- 1. **Les Formateurs**

Pour assurer une telle formation, il serait judicieux de faire appel *(une seule réponse)*

1. ⁪ aux experts locaux uniquement 2. ⁪ aux experts internationaux uniquement 3. ⁪ aux experts locaux et internationaux

4. ⁪ sans opinion

- 1. **Validation des Acquis**

La validation des acquis d’une telle formation exige de : (*plusieurs réponses possibles*)

1. ⁪ réussir à un examen final 2. ⁪ rédiger et défendre un mémoire 3. ⁪ réaliser un projet concret dans le domaine de la protection des droits de l’homme et la santé 4. ⁪ la participation aux activités est suffisante

5. ⁪ Autres ( à préciser)……………………………………………………………………………………………………………………..

6. ⁪ Sans opinion

1. **Observations et commentaires**

Auriez-vous des observations et commentaires sur cette étude. Si oui, merci de les synthétiser dans l’espace réservée ci-dessous

………………………………………………………………………………………………………………………………………………….

………………………………………………………………………………………………………………………………………………….

………………………………………………………………………………………………………………………………………………….

…………………………………………………………………………………………………………………………………………………..

…………………………………………………………………………………………………………………………………………………..

Nous vous remercions de votre collaboration et vous prions de renvoyer le questionnaire dûment rempli à :

Dr. Emmanuel Kabengele Mpinga

Institut de Médecine Sociale et préventive

Faculté de Médecine, Université de Genève

Centre Médical Universitaire

Michel Servet 1, 1211 Genève 4

SUISSE

E-mail : [Emmanuel.kabengele@medecine.unige.ch](mailto:Emmanuel.kabengele@medecine.unige.ch)
